# Thermal, creep-recovery and viscoelastic behavior of high density polyethylene/hydroxyapatite nano particles for bone substitutes: effects of gamma radiation

**DOI:** 10.1186/1475-925X-13-125

**Published:** 2014-08-28

**Authors:** Othman Y Alothman, H Fouad, S M Al-Zahrani, Ayman Eshra, Mohammed Fayez Al Rez, S G Ansari

**Affiliations:** Chemical Engineering Department, Faculty of Engineering, King Saud University, P. O. Box 800, Riyadh, 11421 Saudi Arabia; The Saudi Electronic University, P. O. Box 93499, Riyadh, Saudi Arabia; Applied Medical Science Department, Riyadh Community College, King Saud University, Riyadh, Saudi Arabia; Biomedical Engineering Department, Faculty of Engineering, Helwan University, P. O. Box 11792, Helwan, Egypt; Biomedical Engineering Department, Faculty of Engineering, Minia University, Minia, Egypt; Center for Interdisciplinary Research in Basic Sciences, Jamia Millia Islamia, New Delhi, 110025 India

**Keywords:** HDPE, HA, Nano, Creep, DMA, Gamma radiation

## Abstract

**Background:**

High Density Polyethylene (HDPE) is one of the most often used polymers in biomedical applications. The limitations of HDPE are its visco-elastic behavior, low modulus and poor bioactivity. To improve HDPE properties, HA nanoparticles can be added to form polymer composite that can be used as alternatives to metals for bone substitutes and orthopaedic implant applications.

**Method:**

In our previous work (BioMedical Engineering OnLine 2013), different ratios of HDPE/HA nanocomposites were prepared using melt blending in a co-rotating intermeshing twin screw extruder. The accelerated aging effects on the tensile properties and torsional viscoelastic behavior (storage modulus (G’) and Loss modulus (G”)) at 80°C of irradiated and non-irradiated HDPE/HA was investigated. Also the thermal behavior of HDPE/HA were studied. In this study, the effects of gamma irradiation on the tensile viscoelastic behavior (storage modulus (E’) and Loss modulus (E”)) at 25°C examined for HDPE/HA nanocomposites at different frequencies using Dynamic Mechanical Analysis (DMA). The DMA was also used to analyze creep-recovery and relaxation properties of the nanocomposites. To analyze the thermal behavior of the HDPE/HA nanocomposite, Differential Scanning Calorimetry (DSC) was used.

**Results:**

The microscopic examination of the cryogenically fractured surface revealed a reasonable distribution of HA nanoparticles in the HDPE matrix. The DMA showed that the tensile storage and loss modulus increases with increasing the HA nanoparticles ratio and the test frequency. The creep-recovery behavior improves with increasing the HA nanoparticle content. Finally, the results indicated that the crystallinity, viscoelastic, creep recovery and relaxation behavior of HDPE nanocomposite improved due to gamma irradiation.

**Conclusion:**

Based on the experimental results, it is found that prepared HDPE nanocomposite properties improved due to the addition of HA nanoparticles and irradiation. So, the prepared HDPE/HA nanocomposite appears to have fairly good comprehensive properties that make it a good candidate as bone substitute.

## Introduction

Bioactive nano-ceramic reinforced polymer has been under study as bone substitutes since early 1980s [1–8]. When developing a bone substitute material, its stiffness is considered a crucial aspect. It is known that the bone remodeling is strongly dependent on sufficient and proper loading conditions on the bone. Thus, the load transferred from the implant or bone substitute to the surrounding bones depends on the bone substitute stiffness [9, 10]. Therefore, the replacement of hard tissue requires biocompatible as well as bioactive materials with properties similar to those of natural bone, to avoid the surrounding bone osteoporosis [11].

High Density Polyethylene (HDPE) is one of the most often used polymers in biomedical applications such as bone substitutes and orthopedic prostheses. The limitations of HDPE are its visco-elastic behavior, low modulus and poor bioactivity. Therefore, great efforts have been done to overcome these weaknesses [12–16]. To improve HDPE properties, different types of ceramic nano-particles can be added to form polymer matrix composite that can be used as alternatives to metals for bone substitutes and orthopedic implant applications [16–18]. Different types of ceramic materials such as carbon nanofibers, nano-clay and Hydroxyapatite (HA) have been used as fillers for producing HDPE composites [17–19]. For biomedical application, HA nano-particles have been widely used for improving the mechanical and bioactivity behavior of HDPE. The HA improves the composite stiffness and bioactivity, while HDPE provides toughness. An important design consideration in the long term uses of HDPE, as bone substitutes and orthopedic prostheses, is its integrity under applied load. As maintained earlier, HDPE is a viscoelastic material in nature and its deformation behavior is stress and time dependent. The HDPE viscoelastic and creep behavior easily describe its deformation under load with time and give useful data about the long term integrity of the material in such applications [17–21]. Therefore, the addition of HA nanoparticles as reinforcement may improve both bioactivity, and viscoelastic and creep behavior of HDPE.

Gamma radiation is widely used for sterilizing most of the medical products that made of plastics, because it decomposes the DNA molecules of living organisms. The material has usually been irradiated between 25 and 70 kGy [22, 23]. Nevertheless, this type of sterilization process can produce changes in the molecular structure of the polymer. It has negative effects on the entanglement density of long molecular chains as well as concentration of the tie molecules. On the other hand, the sterilization promotes crosslinking and affects the composite properties [22, 23]. Kane RJ et al. [24] studied the effects of hydroxyapatite (HA) morphology and content on the fatigue behavior of HA reinforced high density polyethylene (HDPE), which suggested that the HA whiskers directly improved the fatigue life and damage tolerance of HA reinforced polymers for synthetic bone substitutes. Soltani Z. et al. [25] studied the effects of irradiation on the mechanical and thermal properties of LDPE/HA powder which was synthesized via hydrolysis method. The samples were subjected to irradiation under 10 MeV electron beam in 75-250 kGy doses. They found that the mechanical and thermal properties more dependent on the hydroxyapatite content than on radiation. Yakov BU. et al. [26] studied the changes in the creep behavior of biaxially oriented LLDPE due to gamma irradiation. Their results showed an improvement in the creep resistance with irradiation does below 4 MR. Yeo S S. and Hsuan YG. [27] studied the tensile creep behavior of polyethylene-terephthalate (PET) and HDPE geogrids. The results showed that the creep deformation of PET geogrid is less than that of HDPE geogrid. The HDPE geogrid exhibited primary, secondary, and tertiary creep stages before rupture, whereas only primary and tertiary creep were observed in the PET geogrid.

From the literature, it can be remarked that a few studies have investigated the effects of gamma radiation dose and HA nano particles percentage on the viscoelastic, creep-recovery and relaxation behavior of HDPE/HA nano composite for biomedical applications. Therefore, the present study is a part of research project that intended to (1) fabricate homogenous HDPE/HA nano-composite, and (2) study the effects of HA nano particles percentage and gamma irradiation dose on the thermal, viscoelastic and creep-relaxation behavior of the HDPE/HA nano-composite.

## Materials and method

The polymeric matrix used in the present work is an injection molding grade of HDPE acquired from the Saudi market. The average molecular weight ranges from 700000 to 800000 g/mol. The reported density is about 0.94 g/cm^3^. High purity spray dried synthetic hydroxyapatite nano particles (Fluidinova, Engenharia de Fluidos, SA, Portugal) were used as reinforcement for HDPE matrix. The typical size of HA nano particle is around 100 nm and the average aggregate size is 2.5 μm. The specific surface area of the HA nano particles is 100 m^2^/g.

HDPE and HA nano particles were first physically mixed according to the desired weight fractions. HDPE/HA ratios of 0%, 10%, 20% and 30% wt were fed on Farrell co-rotating intermeshing twin screw extruder. The screw speed and temperature were kept constant at 12 rpm and 190°C respectively. The strings from the extruder were cooled in a water bath at about 12°C, air dried, then palletized into granules. The granules were further dried and conditioned in the lab environment for 2 days. The granules were then injection molded to get a set of standard ASTM D638 type-I specimens [28]. The nano composite specimens were exposed to gamma irradiation at doses of 0, 35 and 70 kGy at rate of 5 kGy/hr at room temperature in vacuum.

The cryo-fracture surfaces of HDPE/HA nano composites were examined using Scanning Electron Microscope (SEM Model ISM 6360A, Jeol Company, Japan) at 20 KV. The specimens were fixed with double coated carbon tape. All the specimens were coated with Platinum before scanning to dissipate the build-up of heat and electrical charges using SEM using JEOL, IFC 1600 coating machine. The melt flow index (MFI) test was performed on a Ray-Ran advanced melt flow system following ASTM D-1238 at 190°C and load of 2.16 Kg.

Calorimetric measurements for neat HDPE and its nanocomposite specimens were performed using Differential Scanning Calorimetry (DSC-7 series, Thermal Analysis System, Perkin Elmer, Covina, California, USA). Each specimen (5–7 mg) was sealed in an aluminum pan and heated from 25 to 145°C at rate of 5°C/min and then cooled down to 25°C at cooling rate of 5°C/min. The heat of melting was calculated through the integration of the area under the DSC endothermic peak of the DSC thermogram. The melting temperatures of the blends were taken at the peaks of the melting processes. The percentage crystallinity was calculated by normalizing the heat of melting to that of 100% crystalline PE (290 J/g) [21, 25–27]. Thermogravimetric analysis (TGA) of HDPE/HA was conducted using TA instrument (Q500 TGA, United States). The precisely weighed specimen was heated to 550°C at a rate of 10°C/min under nitrogen flow rate of 35 ml/min.

The tensile storage and loss modulus (viscoelastic behavior) of irradiated and non-irradiated HDPE/HA nano-composites specimens were characterized using standard Dynamic Mechanical Analysis Machine (DMA Model 2980TA Instrument, New Castle, Delaware, USA). The specimens were tested over a frequency range from 0.01 to 100 Hz at temperature 25°C. The DMA was also used to measure the creep-recovery and relaxation behavior HDPE/HA nano-composites specimens. The creep behavior of HDPE/HA specimens was obtained by applying constant stresses of 2, 5, 10 MPa for 8 hrs at room temperature. The recovery behavior was measured for 8 hrs after removing the applied stress.

The DMA machine was also used for measuring the relaxation behavior of HDPE/HA specimens. During the relaxation tests different HDPE/HA nano-composite specimens were subjected to a pre-determined strain level of 3% that was held constant while the decaying in the stress with time was recorded during test period of 3 hrs at room temperature. All the experiments were repeated at least three times, conducted in triplicate (n = 3) and error bars represent standard deviations (SD). All the values were represented as means ± SD.

## Results and discussions

### Morphological examination

**Figure 1:**
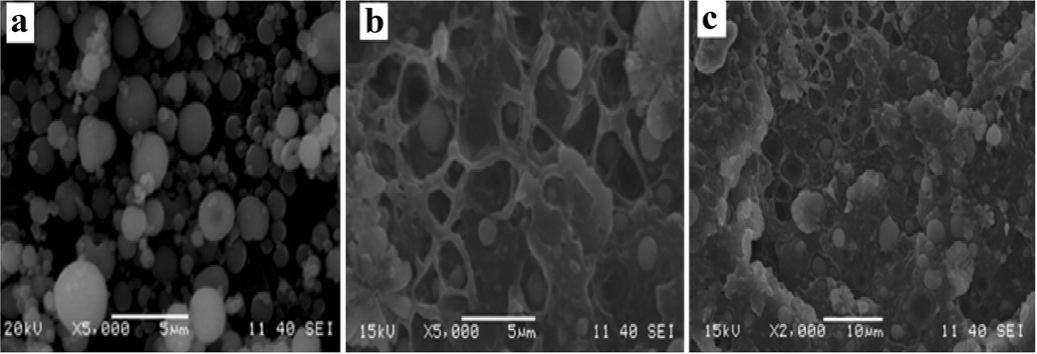
**SEM micrograph of (a) HA nanoparticles (b) HDPE/HA nano composite 10% and (c) 30%.**

The cryo-fracture surface morphologies of HA nanoparticles and HDPE/HA (10%, 30%) are shown in Figure 1a, b and c respectively. A good distribution of HA nano particles, appear as bright spots in the dark HDPE matrix, was achieved. In spite of the HA particles were in the nano-range according to the manufacturer specifications, the cryo-fracture surface morphologies shows remarkable agglomeration of HA nano particles. This agglomeration could be attributed to the tendency of HA nano particles to decrease their contact surfaces with HDPE matrix.

### Melt flow index (MFI)

**Figure 2:**
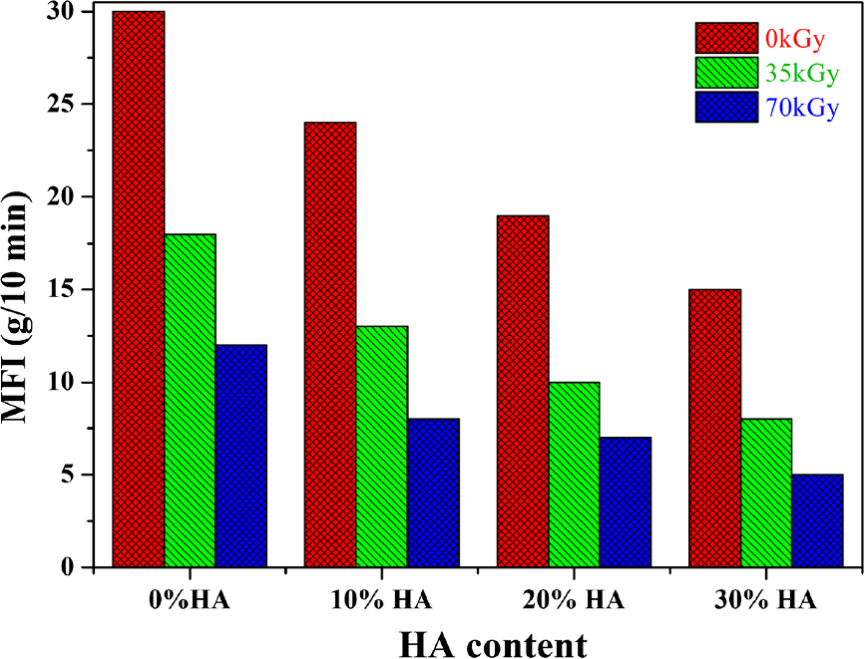
**Effects of HA and Gamma radiation on the HDPE melt flow index (MFI).**

The effects of HA and Gamma irradiation on the HDPE/HA MFI is shown in Figure 2. As can be observed, the MFI of HDPE/HA strongly decreases with gamma irradiation and HA nanoparticles content. This reduction in the HDPE nanocomposite MFI due to irradiation can be attributed to the chain crosslinking. On the other hand when HA is added to the polymer, the material viscosity increases and hence resistance to flow increases. Similar results have been also obtained where the MFI decreases due to irradiation and presence of HA for HDPE/HA (20:80) [29].

### Calorimetric measurements

**Figure 3:**
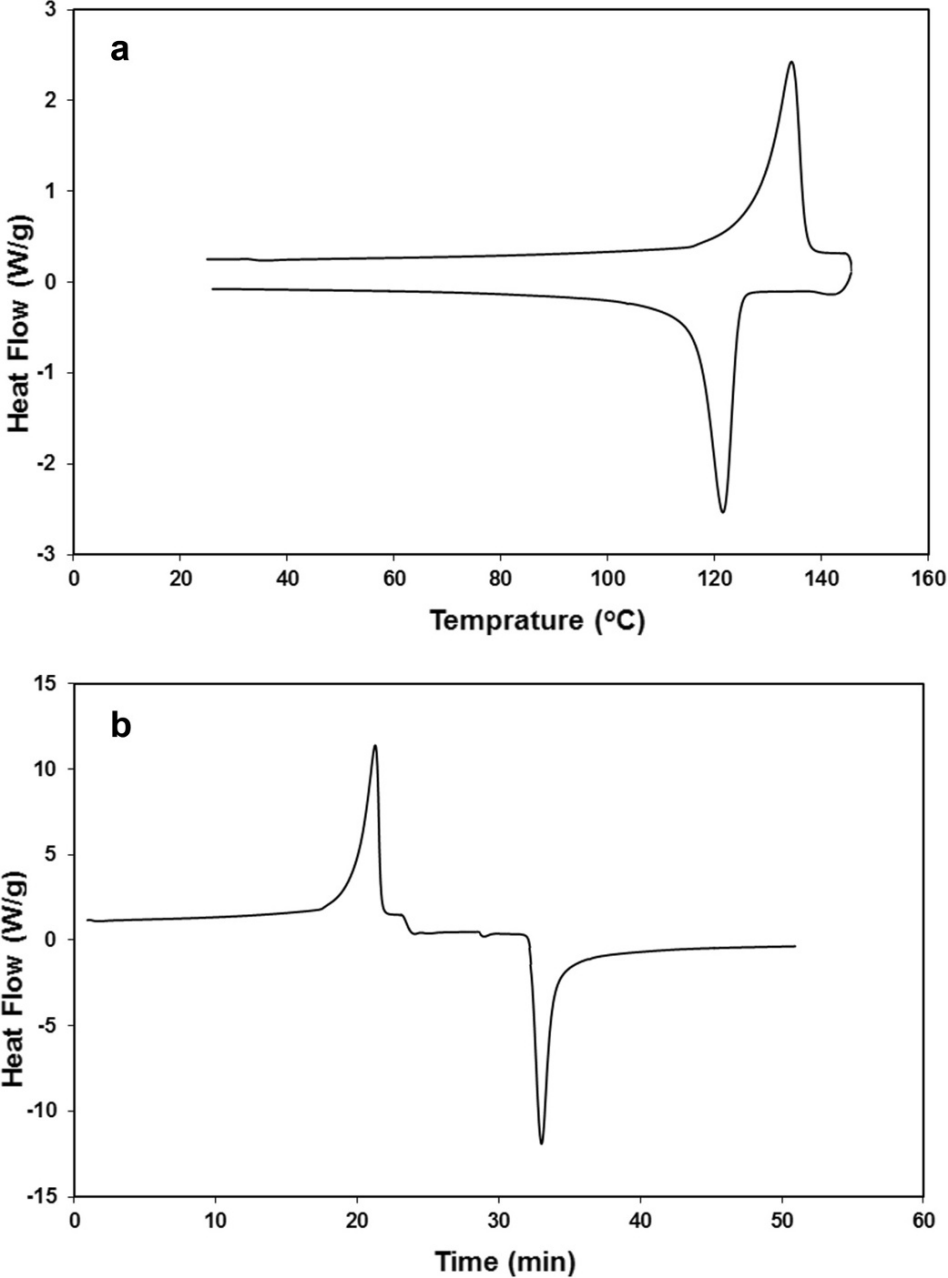
**Heat–cooling processes for irradiated (70 kGy) HDPE/HA (30%) nanocomposite (a) heat flow against temp (b) heat flow against time.**

**Figure 4:**
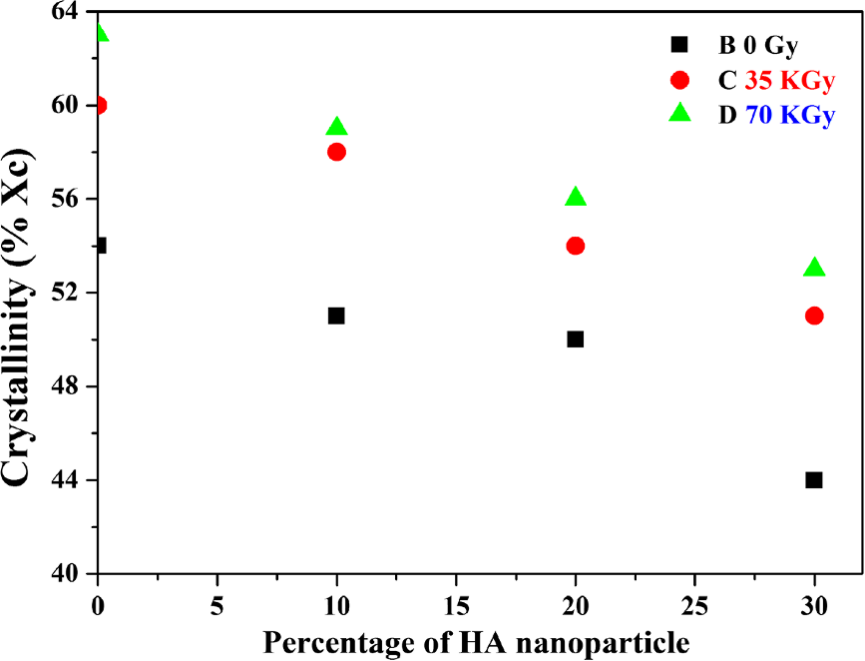
**Effects of HA and Gamma radiation on the HDPE crystallinity.**

**Figure 5:**
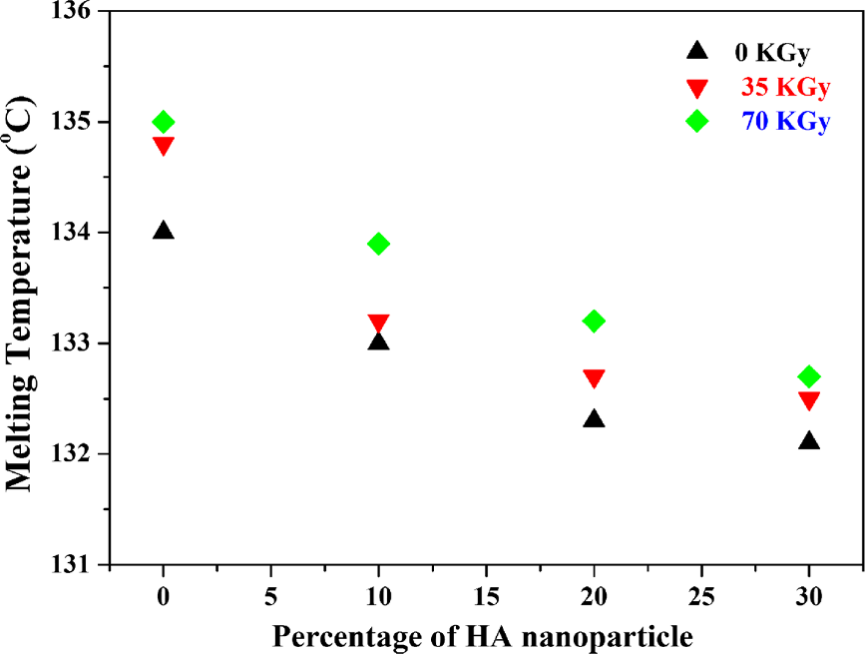
**Effects of HA and Gamma radiation on the HDPE melting temperature.**

The thermal properties for the melting and crystallization of irradiated (70 kGy) HDPE/HA (30%) nanocomposite is shown in Figure 3. This figure shows the overlay DSC curves as a function of temperature and time. The degree of crystallinity and melting temperature of the first heat for irradiated and non-irradiated neat HDPE and HDPE/HA nanocomposites are shown in Figures 4 and 5 respectively. The results indicated that the degree of crystallinity (X_c_) decreased with increasing the HA nanoparticle ratio. Figure 4 indicated that the X_c_ of neat HDPE decreased by 19% due to the addition of 30% HA to the polymeric matrix. This figure also indicated that the X_c_ of neat HDPE and its nanocomposites increased due to gamma irradiation. The crystallinity of HDPE/HA (30%) increased by 20% due to irradiation with 70 kGy. The reduction on the HDPE/HA nanocomposite crystallinity can be attributed to the formation of HA nanoparticles conglomerates in the HDPE matrix. At higher ratios of fillers these conglomerates exceed the critical size and lose their nucleating capacity. Also the presence of HA nanoparticles restricted the mobility of the molecules and hence the crystallinity decreased. At high ratio of HA nanoparticles, the degree of crystallinity decreased not only due to conglomeration of fillers but also due to confinement and entanglement effects. Moreover, the effect of nanoparticles on polymer crystallization is strongly affected by the shape and ratio of nanoparticles [17–19, 30]. On the other hand, the HA filler has higher specific heat capacity that make it a better heat conductor resulting in faster cooling of HDPE/HA nano-composite. This high rate of cooling resulted in thin lamellar formation rather than thick crystal growth leading to lower degree of crystallinity [30, 31]. This decrease in the crystallinity has negative effects on the polymer mechanical properties where the higher degree of crystallinity leads to stiffer and stronger composite. However, for the HDPE/HA nanocomposite, these negative effects can be negated by the presence of HA nanoparticles which stiffen the polymer. The DSC results also indicated that the crystallinity of neat HDPE and HDPE/HA nano-composites increased due to irradiation. Such increase of crystallinity can be attributed to the crosslinking of polymer chains due to gamma irradiation. The DSC results also indicated that the melting temperature is slightly changed due to the addition of HA nanoparticles and gamma radiation as indicated in Figure 5.

### Thermogravimetric analysis (TGA)

**Figure 6:**
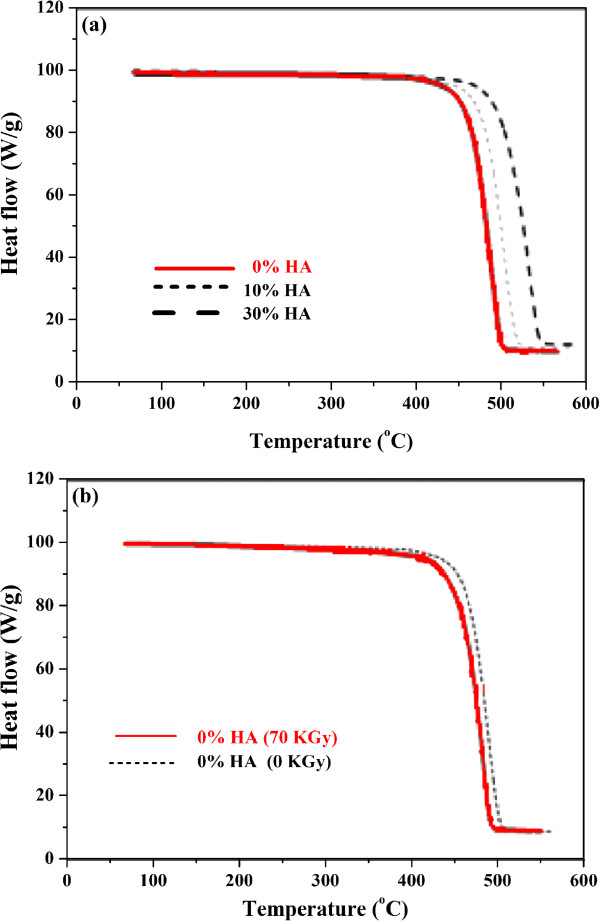
**Thermogravimetric analyses of HDPE and its nanocomposite (a) Effect of HA ratio (b) Effect of Irradiation Dose.**

Figure 6a graphically displays the thermogravimetric analysis of neat HDPE and its nanocomposite. The weight loss vs. temperature profile of neat HDPE and its nanocomposites showed that the weight of these materials remains unchanged until the temperature reached 400°C. These results showed that the weight loss mainly occurred in the range of 420-480°C, 435-520°C and 450-540°C for neat HDPE, 10% HA and 30% HA, respectively, with negligible change at temperature higher than 550°C. This weight loss indicated that the decomposition temperature of HDPE/HA nanocomposites is higher than that of the neat material. These results suggested that the HA nanoparticles confers thermal stability to the HDPE matrix and this can be attributed to good dispersion of the HA nanoparticles. This phenomenon resulting in a delay in the polymer thermo-degradation process. Similar results have been obtained by Bikiaris B. [32] who studied the effects of inorganic nanofillers on the thermal stability of polymers. Bikiaris attributed the increase of polymer thermal stability to many reasons such as shielding effects, gas impermeability of inorganic nanofillers that inhabit the formation and escape of volatile by-products during the degradation, reducing polymer chain mobility and then delaying the composite degradation. The irradiated specimen showed higher thermal stability than that of non-irradiated specimen, as shown in Figure 6b. It can be seen that the weight loss of irradiated specimens remained unchanged until the temperature reaches 433°C. This weight loss mainly occurred in the range of 433-495°C with negligible change at temperature higher than 505°C. Also, the improvements in the nanocomposite thermal stability due to irradiation can be attributed to the reduction of chain mobility which occurred as a positive effect due to gamma irradiation [29].

### Tensile storage and loss modulus E’, E”

**Figure 7:**
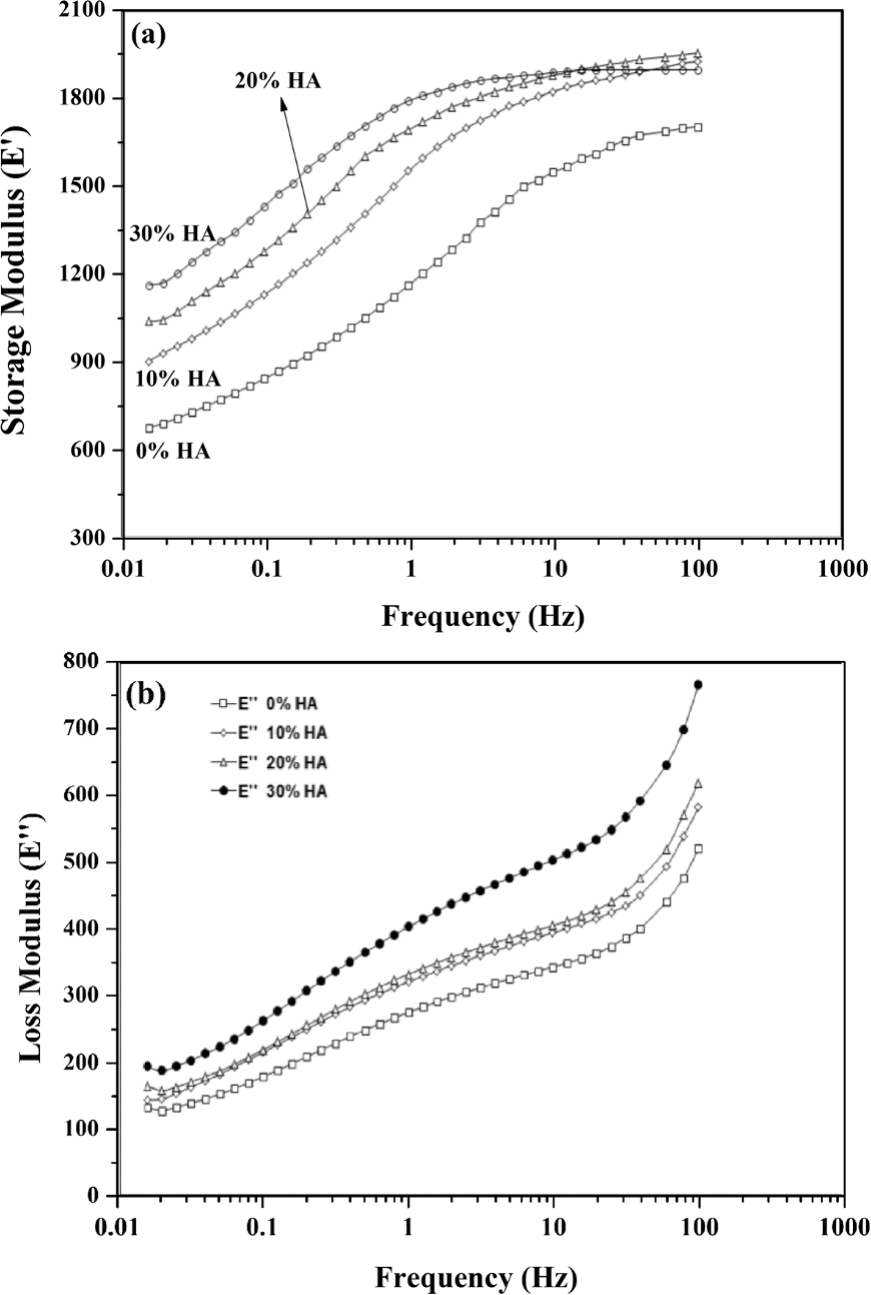
**Effect of HA contents on the (a) storage and (b) loss modulus of HDPE nanocomposite.**

The variations of tensile dynamic mechanical properties (storage modulus E’ and loss modulus E”) of neat HDPE and its HA nanocomposites with frequency are presented in Figure 7. The measurements were carried out at frequencies ranging from 0.01 to 100 Hz at room temperature (25°C). The results showed a strong dependence of the tested material’s viscoelastic behavior on the testing frequency (loading rate), where the storage and loss moduli increased significantly with increasing the testing frequency. These results confirmed that the viscoelastic behavior of HDPE is strain rate dependent. It is noticed that E‘ is more than E” for all types of HDPE specimens which indicated that the elastic behavior of the material is dominant over the viscous one. Additionally, the storage and loss moduli (E’, E”) of all nano-composites increased with increasing the HA nanoparticles. For example, at 1 Hz, the storage modulus increased from 1.16 to 1.55, 1.69 and 1.79 GPa due to the addition of 10, 20 and 30% HA nanoparticles compared to neat HDPE. For loss modulus, its value increased from 275 to 320, 332 and 403 MPa due to the addition of 10, 20 and 30% HA nanoparticles compared to neat HDPE at the same testing conditions. This increase in the moduli of nanocomposites can be attributed to the increase in the stiffness of polymeric matrix as a result of the decrease of free volume and the mobility restriction due to the presence of HA nanoparticles [17–19].

**Figure 8:**
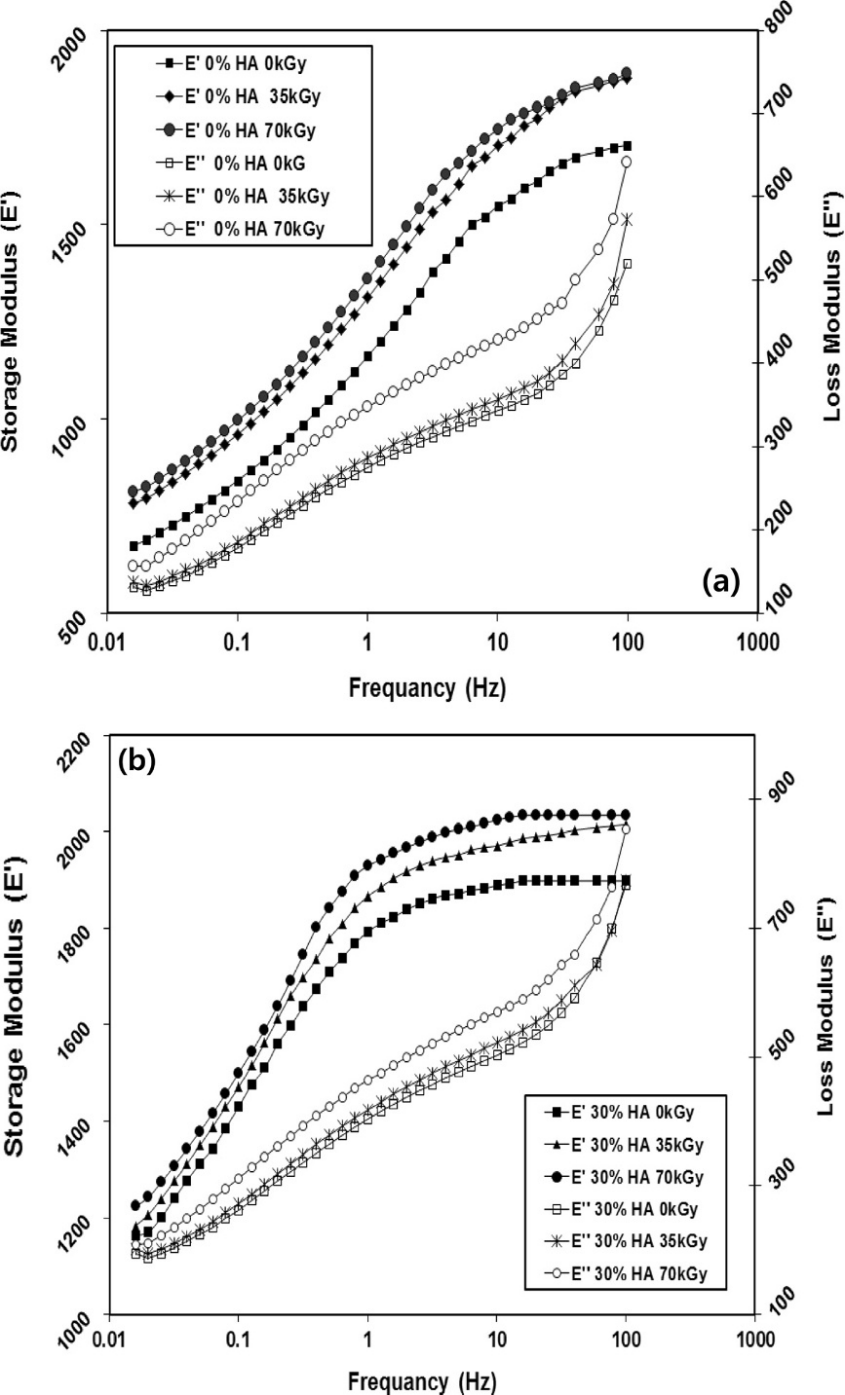
**Effect of Gamma radiation on the storage and loss modulus for (a) HDPE and (b) HDPE/HA.**

The effects of gamma irradiation dose on the storage and loss moduli of neat HDPE and its nanocomposite as a function of testing frequency are shown in Figure 8. The results indicated that the storage and loss modulus significantly increased with increasing the irradiation dose. For example, at 1 Hz, the storage modulus increased from 1.16 to 1.31 and 1.36 GPa due to the irradiation with 35 and 70 kGy compared to non-irradiated neat HDPE. For loss modulus, its value increased from 275 to 300 and 350 MPa due to irradiation with 35 and 70 kGy compared to non-irradiated neat HDPE at the same testing conditions. The improvement in the viscoelastic behavior due to gamma irradiation can be attributed to the crosslinking of HDPE chains that occurred in the amorphous region resulting in creation of rigid regions HDPE matrix [31].

### Creep-recovery behavior

**Figure 9:**
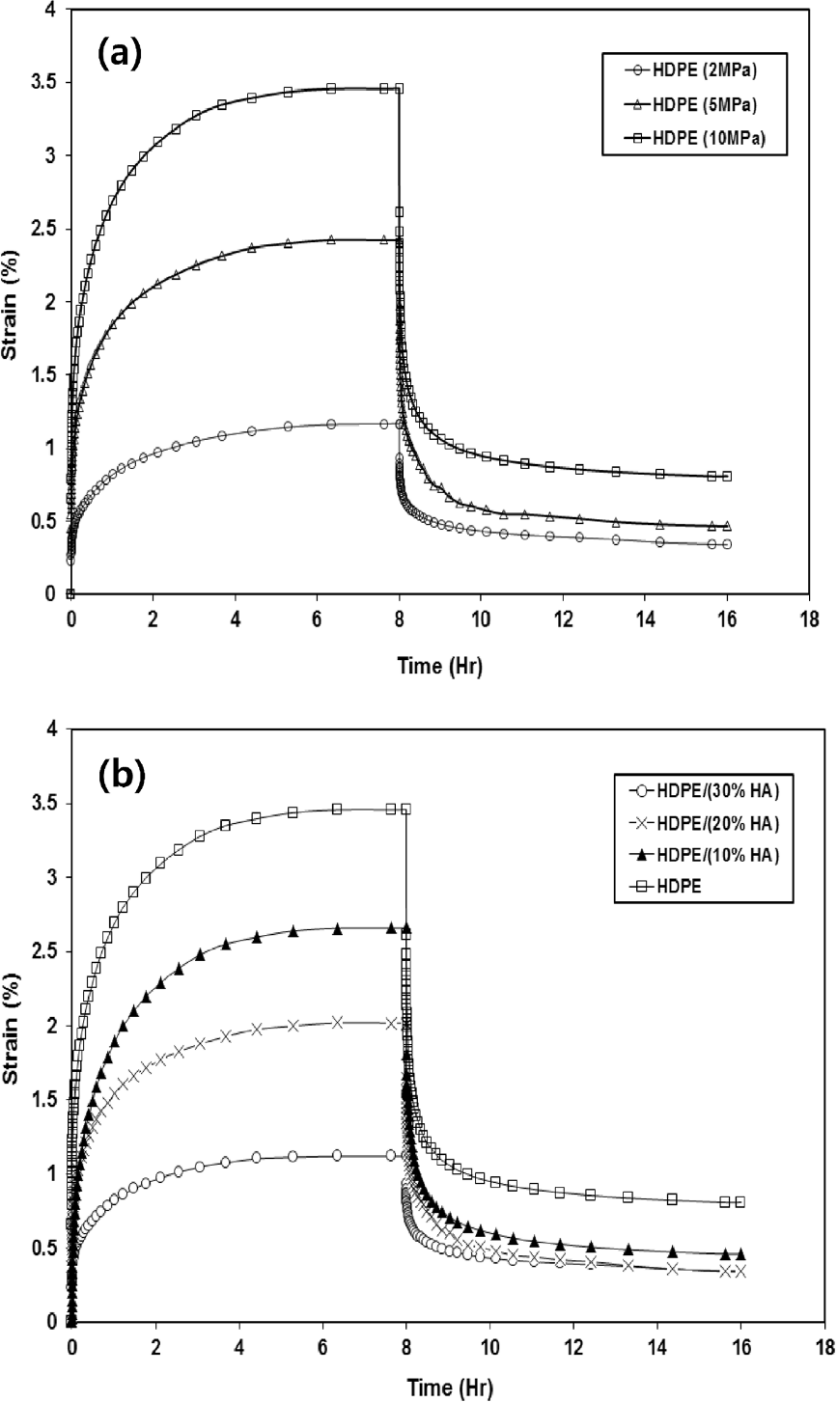
**Creep-recovery response of (a) HDPE at different loads and (b) HDPE/HA.**

**Figure 10:**
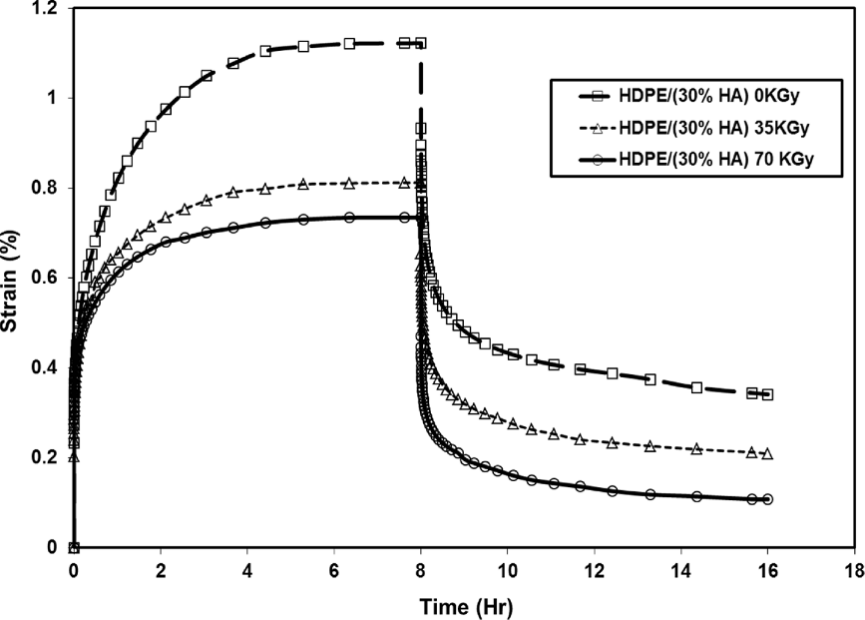
**Effects of Gamma irradiation dose on the creep-recovery resistance of HDPE/HA (30%).**

Figure 9 shows the creep-recovery response of neat HDPE and its nanocomposites at different loading conditions. The tests were carried out at stress levels of 2, 5, 10 MPa at a temperature of 25°C. The results showed an initial elastic deformation and subsequently, the strain slowly increased as long as the stress is maintained. After stress removal (at 8 hrs of loading), the elastic strain is quickly recovered, and the portion of the creep strain is recovered slowly in the course of time. The creep test results indicated that the creep strain for neat HDPE and its nanocomposite increases (or the creep resistance decreases) with increasing the applied stress as expected. Moreover, the creep resistance increased significantly with the addition of HA nanoparticles. The addition of 10%, 20% and 30% HA increased the creep resistance of HDPE nanocomposites by 25%, 41% and 67% respectively comparing to the neat HDPE. Conversely, the remaining residual strain decreased by 42%, 57% and 57% respectively. The improvement of creep resistance due to the addition of HA nanoparticles to HDPE matrix can be attributed to the improvement of polymeric matrix stiffness due to the reduction of free volume and the chains mobility restriction [17–19]. Figure 10 shows the effects of gamma irradiation dose on the creep-recovery resistance of HDPE/HA (30%) as a function of time. The results indicated that the creep resistance increases due to irradiation and the remaining residual strain decreased. Irradiation improved the creep behavior of HDPE/HA nanocomposites due to chain crosslinking that occur in the amorphous region resulting in rigid regions in the polymer matrix.

### Relaxation behavior

**Figure 11:**
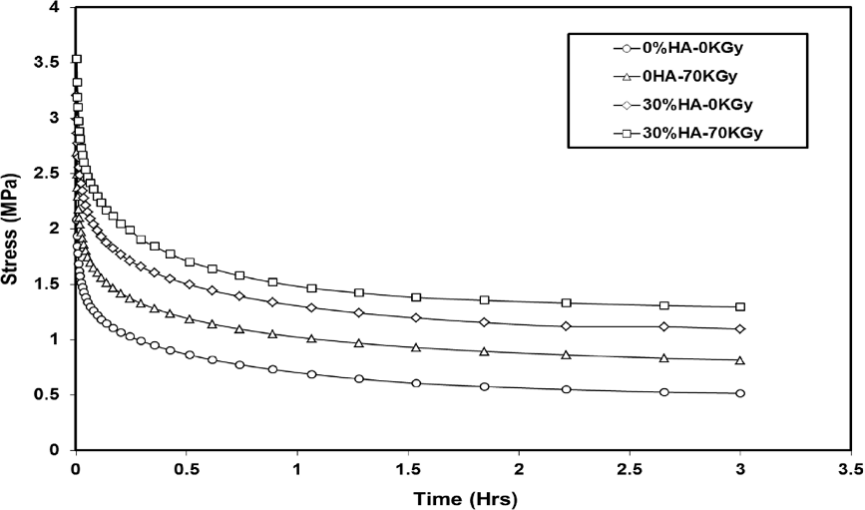
**Stress relaxation behavior of irradiated and non-irradiated HDPE and its nanocomposite.**

The relaxation test for neat HDPE and its nanocomposite specimens were carried out at predetermined strain level of 3%. The applied strain was held constant while the decaying in the stress with time was recorded for a period of 3 hrs. The tests were conducted under a constant temperature of 25°C. Figure 11 shows the stress relaxation graphs of the irradiated and non-irradiated neat HDPE and its 30% HA nanocomposite. The plots showed that, the relaxation stress levels of HDPE/HA nanocomposites and irradiated specimens are higher than that for neat HDPE specimens, over the whole test duration. Furthermore, the initial and remaining relaxation stresses also have higher values compared to non-irradiated neat HDPE specimens. The increase of the initial stress can be attributed to the changes in nanocomposite stiffness due to the presence of HA nanoparticles in the polymer matrix. With reference to Figure 11, it is clear that the relaxation stress value of irradiated HDPE/HA (30%) specimens after 3 hrs reduced to 37% of its initial value through the relaxation test, while it dropped to 24% of initial value for neat HDPE specimens. The value of stress reduction occurred in HDPE/HA specimens is higher than that on of neat HDPE, indicating that in the biomedical applications (bone substitutes and plates, hip joint cup etc), HDPE/HA nanocomposite would transfer a large amount of stress to the surrounding bone (reduce stress shielding). This phenomenon will lead to an improvement in the artificial joint performance [33].

## Conclusions

Commercially available hydroxyapatite nanoparticles (~100 nm size and specific surface area of 100 m^2^/g.) were used as reinforcement of locally available HDPE with an average molecular weight of 700,000 to 800,000 g/mol in a different ratios of 0%, 10%, 20% and 30% wt. After twin screw extrusion and injection molding, a good distribution of HDPE and HA nanoparticles was successfully prepared and exposed to gamma irradiation at doses of 0, 35 and 70 kGy at a rate of 5 kGy/hr, at room temperature It is found that melting temperature was insignificantly affected by the addition of HA nanoparticles while the crystallinity of HDPE composites reduced due to the restriction of mobility of the molecules. The tensile storage and loss moduli, and creep resistance of the nanocomposites increased proportionally with the HA particle content. Irradiation of HDPE and its nanocomposites resulted in an increase of the tensile storage and loss moduli, creep recovery and resistance. In conclusion it was found that increasing the HA content and irradiation improved the relaxation behavior of HDPE and its nanocomposites significantly.
